# *Astragalus* and its formulas as a therapeutic option for fibrotic diseases: Pharmacology and mechanisms

**DOI:** 10.3389/fphar.2022.1040350

**Published:** 2022-11-03

**Authors:** Yi Zhu, Yilu Chai, Guojin Xiao, Yufei Liu, Xiaohong Xie, Wei Xiao, Pengcheng Zhou, Wei Ma, Chuantao Zhang, Liuying Li

**Affiliations:** ^1^ Department of Respiratory, Hospital of Chengdu University of Traditional Chinese Medicine, Chengdu, China; ^2^ Nursing Department, Hospital of Chengdu University of Traditional Chinese Medicine, Chengdu, China; ^3^ Chengdu University of Traditional Chinese Medicine, Chengdu, China; ^4^ Department of Heart Disease of Traditional Chinese Medicine, Zigong First People’s Hospital, Zigong, China

**Keywords:** *Astragalus mongholicus* Bunge, active components, fibrosis, anti-fibrosis effects, traditional Chinese medicine

## Abstract

Fibrosis is the abnormal deposition of extracellular matrix, characterized by accumulation of collagen and other extracellular matrix components, which causes organ dysfunction and even death. Despite advances in understanding fibrosis pathology and clinical management, there is no treatment for fibrosis that can prevent or reverse it, existing treatment options may lead to diarrhea, nausea, bleeding, anorexia, and liver toxicity. Thus, effective drugs are needed for fibrotic diseases. Traditional Chinese medicine has played a vital role in fibrotic diseases, accumulating evidence has demonstrated that *Astragalus* (*Astragalus mongholicus* Bunge) can attenuate multiple fibrotic diseases, which include liver fibrosis, pulmonary fibrosis, peritoneal fibrosis, renal fibrosis, cardiac fibrosis, and so on, mechanisms may be related to inhibition of epithelial-mesenchymal transition (EMT), reactive oxygen species (ROS), transforming growth factor beta 1 (TGF-β1)/Smads, apoptosis, inflammation pathways. The purpose of this review was to summarize the pharmacology and mechanisms of *Astragalus* in treating fibrotic diseases, the data reviewed demonstrates that *Astragalus* is a promising anti-fibrotic drug, its main anti-fibrotic components are Calycosin, Astragaloside IV, *Astragalus* polysaccharides and formononetin. We also review formulas that contain *Astragalus* with anti-fibrotic effects, in which *Astragalus* and *Salvia miltiorrhiza* Bunge, *Astragalus* and *Angelica sinensis* (Oliv.) Diels are the most commonly used combinations. We propose that combining active components into new formulations may be a promising way to develop new drugs for fibrosis. Besides, we expect *Astragalus* to be accepted as a clinically effective method of treating fibrosis.

## Introduction

Fibrosis may be caused by normal healing or by pathological deposition of fibrous connective tissue (Zhang and Zhang, 2020), which occurs in various organs such as lung, heart, kidney, peritoneum, and is responsible for approximately 45% of all deaths in western developed countries (Wynn, 2004). Owing to inappropriate assignment, adequate epidemiological evidence is lacking (Asrani et al., 2019; Sgalla et al., 2019), available data highlights that up to 3 million people worldwide are affected by idiopathic pulmonary fibrosis (Martinez et al., 2017). More than 1 million people died for liver cirrhosis in 2010 (Mokdad et al., 2014), nearly all forms of heart diseases lead to cardiac fibrosis (Czubryt and Hale, 2021), 10% of the world’s population suffer from chronic kidney disease and renal fibrosis (Humphreys, 2018), indicating a high prevalence of fibrotic diseases.

The process of fibrosis is dynamic and occurs as a reaction to repeated or chronic tissue injuries. Trauma, toxic, drug-induced, infectious, or autoimmune injuries can all contribute to fibrosis (Thannickal et al., 2004). Inflammation is the most common precipitating factor (Aydın and Akçalı, 2018), however, in some cases removing the trigger does not stop the fibrosis process (Roehlen et al., 2020). Even though fibrosis plays a pivotal role in restoring normal tissue architecture, relentlessly progressive and irreversible fibrosis caused by repetitive or severe injury may lead to organ dysfunction and ultimately organ failure (Henderson et al., 2020).

Although understanding of the pathogenesis and management of fibrotic diseases have been greatly improved, there’s currently no cure for them (Martinez et al., 2017; Nastase et al., 2018; Roehlen et al., 2020; Czubryt and Hale, 2021). What’s more, current treatments may lead to diarrhea, nausea, bleeding, anorexia, and liver toxicity (Martinez et al., 2017; Richeldi et al., 2017). Thus, the main aim of treatment is to relieve symptoms as much as possible and slow down fibrosis progression (Martinez et al., 2017). Therefore, alternative approaches are urgently needed, natural products have always held a privileged position as valuable sources and inspirations for new drug development (Newman and Cragg, 2020). Some Chinese medicines show promising anti-fibrotic effects (El-Tantawy and Temraz, 2022; Zhang et al., 2022a).

*Astragalus* (*Astragalus mongholicus* Bunge), known as *Huangqi* in China, has a long history of medicinal use for more than 2000 years (Bi et al., 2020). In Chinese Pharmacopoeia, the most commonly used are *Astragalus membranaceus* (Fisch.) Bge. and *Astragalus membranaceus* (Fisch.) Bge. Var. mongholicus (Bge.) Hsiao. As an adaptogenic herb, *Astragalus* holds an important place in traditional Chinese medicine and is a popular herbal medicine worldwide. The medicinal value of *Astragalus* was first recorded in Wu shi er bing fang (Formularies for 52 Kinds of Disorders) (Gu et al., 2018). While in Shennong’s Materia Medica Classic (200–300 AD, Han Dynasty) (Zhao et al., 2022), *Astragalus* was classified as top grade and used for “tonifying deficiency”, which means *Astragalus* has fewer side-effects and excellent clinical efficacies (Bi et al., 2020). *Astragalus* has been widely used in foods and clinics, it is one of the approved medicine food homology species in China (Li et al., 2020). Traditionally, *Astragalus* is used to improve the body’s vital functions in chronic disease patients and healthy persons (Liu et al., 2017). More importantly, “deficiency” is the major initiator of fibrosis. Thus, *Astragalus* is commonly used for fibrosis treatment (Yao and Jiang, 2003; Xu and Liu, 2020; Sun et al., 2022). *Astragalus* contains more than 200 compounds, including triterpene saponins, flavonoids, and polysaccharides (Shan et al., 2019). Pharmacological studies have shown that *Astragalus* has anticancer, anti-aging, anti-oxidation, anti-photoaging, anti-inflammation, and improvement of cardiomyocyte functions (Li et al., 2014a; Liu et al., 2017).

In recent years, numerous studies have demonstrated the anti-fibrotic properties of *Astragalus* and its active components, including pulmonary, cardiac, liver, renal and peritoneal fibrosis. Mechanisms may be related to the inhibition of epithelial-mesenchymal transition (EMT), reactive oxygen species (ROS), transforming growth factor beta 1 (TGF-β1)/Smads, apoptosis, and inflammation pathways (Yu et al., 2016; Qian et al., 2018; Zheng et al., 2021; Li et al., 2022). In this review, we summarized *Astragalus*’s antifibrotic effects and mechanisms to provide a reference for the follow-up studies.

## Materials and methods

An online literature search was carried out at PubMed, Web of Science, Google Scholar, and China National Knowledge Infrastructure, covering 2012 until April 2022. The following keywords were used: “*Astragalus*” and “fibrosis”, “pulmonary fibrosis”, “liver fibrosis”, or “renal fibrosis”, “cardiac fibrosis”, and “peritoneal fibrosis”. The references of all retrieved articles were also reviewed to include relevant literature.

## Myofibroblast activation in organ fibrosis

Myofibroblasts are cells that produce collagens and are involved in the fibrosis of different tissues, which are gradually activated by inflammatory and mechanical conditions (Pakshir et al., 2020). The origins of myofibroblasts are extensive and incompletely elucidated, including fibroblasts, endothelial cells, pericytes, and bone marrow-derived cells (Yuan et al., 2019). Since TGF-β1 can be activated in inflammatory and mechanical conditions and active TGF-β1 leads to myofibroblast activation, this signal is involved in fibrosis of almost all tissues and is a common signaling pathway in many organs (Pakshir et al., 2020). In some organs, such as the lung, kidney, and peritoneum, EMT is also involved in fibrosis process (Humphreys, 2018; Balzer, 2020; Muthuramalingam et al., 2020).

Activated hepatic stellate cells are the major effectors during hepatic fibrosis (Tsuchida and Friedman, 2017). Multiple signaling pathways regulate them, including platelet-derived growth factor (PDGF) signaling and TGF-β1. After being stimulated, activated hepatic stellate cells transform into myofibroblasts (Aydın and Akçalı, 2018), which secrete collagens I, II, and fibronectin (FN) and lead to liver fibrosis (Brown et al., 2006; Dewidar et al., 2019).

Pulmonary fibrosis can be induced by EMT, myofibroblast activation, and mechanical tension (Thannickal et al., 2004; Yang et al., 2020). Mechanisms include TGF-β1, sonic hedgehog, Notch, Wnt, fibroblast growth factor, and PDGF (Chanda et al., 2019). Common extracellular matrix components (ECM) are collagens I, III, and VI (Deng et al., 2020).

Long-term peritoneal dialysis causes fibrosis and inflammation in the peritoneal membrane. These two processes are frequently bidirectional (Zhou et al., 2016a). In addition, EMT, TGF-β1, and mechanical tension also induce peritoneal fibrosis (Balzer, 2020; Terri et al., 2021).

In kidney, myofibroblast activation can be induced by TGF-β1 and Notch pathway upregulation (Humphreys, 2018). Inflammatory cells such as macrophages and obstructive uropathy can active TGF-β1 to induce renal fibrosis (Gu et al., 2020; Yoon et al., 2020). Common ECM are collagen I, III, V, VI, VII, XV, and FN (Genovese et al., 2014).

Cardiac fibrosis is often caused by inflammation and overexpansion, both of these factors activate myofibroblasts, which secrete ECM such as collagen I, III, and IV (Yuan et al., 2019; Lafuse et al., 2020; Liu et al., 2021a).

## Astragalus and its active antifibrotic components

**FIGURE 1:**
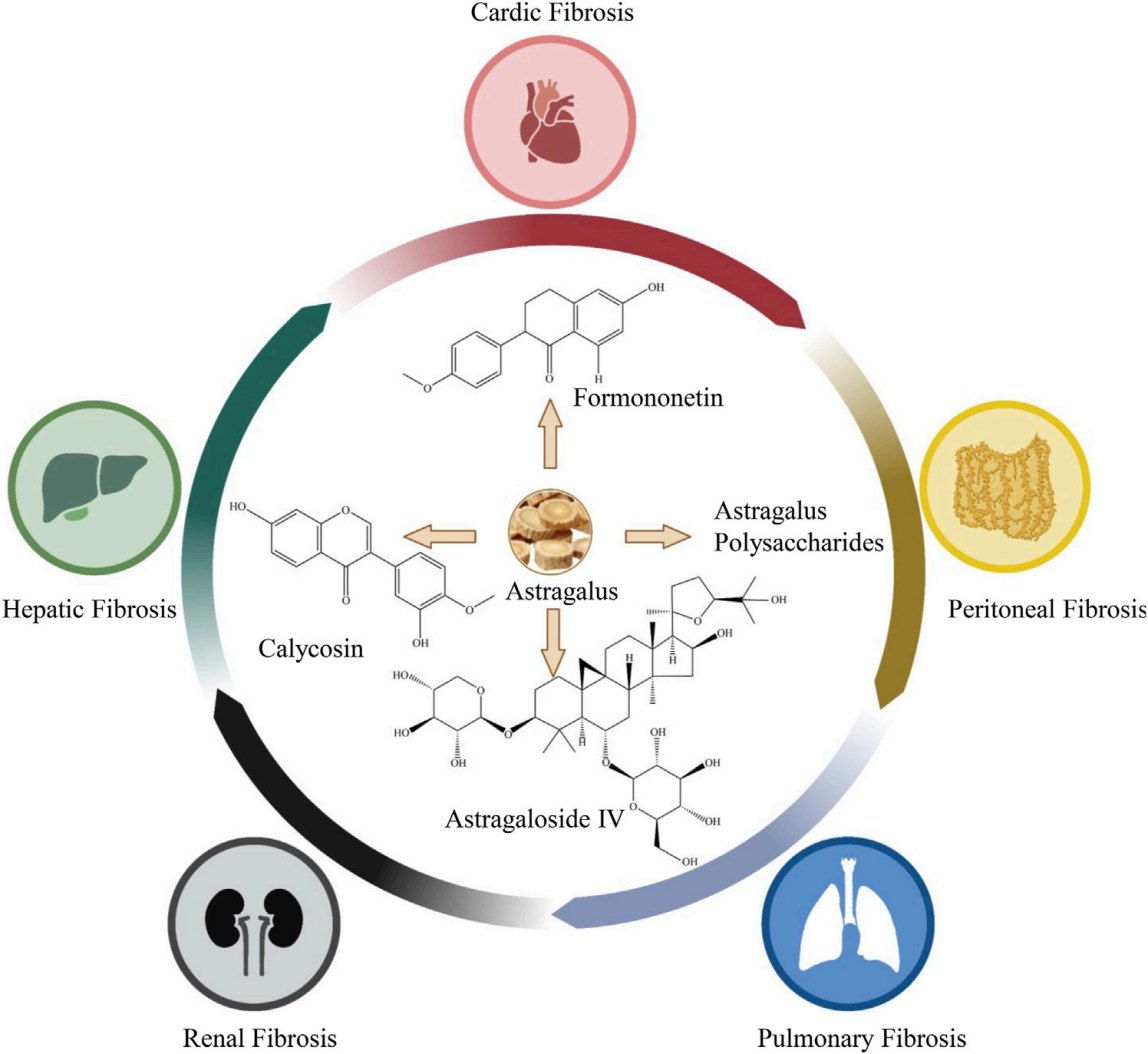
Antifibrosis effects of *Astragalus* and its main active anti-fibrotic components.

*Astragalus* is shown to exert multiple antifibrosis effects (Table 1), and the active antifibrotic components are Calycosin (C16H12O5), Astragaloside IV (C41H68O14), *Astragalus* polysaccharides, and formononetin (C16H12O4) (Figure 1).

**TABLE 1:** Effect of *Astragalus* and its active components on fibrotic diseases.

Disease	Animals/cell lines	Components	Dose	Duration	Mechanisms	References
Liver fibrosis	HSC-T6 cells	Astragaloside IV	20, 40 μg/ml	48 h	activating the NF-κB pathway to inhibit PDGF-BB	Chen et al. (2019)
	Rats	Astragaloside	164 mg/kg	3 w	inhibition of Notch signaling activation	Yongping et al. (2015)
	Liver sinusoidal endothelial cells	*Astragalus* polysaccharides	12.5–200 μg/ml	24 h	increased Young’s modulus of liver sinusoidal endothelial cells	Lu et al. (2018)
	C57BL/6 mice	Calycosin	40 mg/kg, 80 mg/kg	8 w	oxidative stress↓, MMP-1↑/TIMP-1↓, JAK2↑/STAT3↑	Zhang et al. (2021a)
	C57BL/6 mice	Calycosin	12.5, 25, 50 mg/kg/d	4 w	activating farnesoid X receptor	Duan et al. (2017)
Pulmonary fibrosis	Rats	Astragaloside IV	20 mg/kg	14 d	TNF-α↓, IL-6↓; TGF-β1/PI3K/AKT↓/Foxo3a↑/EMT↓	Qian et al. (2018)
	Sprague-Dawley rats	*Astragalus* injection	8 g/kg	28 d	Jagged1/Notch1//TGF-β1↓	Zhou et al. (2016b)
	C57Bl/6J mice	Astragaloside IV	20 mg/kg	14 d	sirt1 AS↑/sirt1↑/AKT↓/Foxo3a↑/EMT↓	Qian et al. (2020)
	RLE-6TN cells		0–200 μg/ml	48 h		
	C57BL/6 mice	Calycosin	7, 14 mg/kg	3 w	AKT/GSK3β/β-catenin/EMT↓	Liu et al. (2021b)
	Sprague-Dawley rats	Astragaloside IV	10, 20, 50 mg/kg	28 d	inhibiting oxidative stress and inflammatory response	Yu et al. (2016)
	C57BL/6 mice	*Astragalus* polysaccharides	200 mg/kg/day	4 w	EMT↓, NF-κB pathway activation↓	Zhang et al. (2020a)
Peritoneal fibrosis	Sprague-Dawley rats	*Astragalus* total saponins	20, 40 mg/kg/day	14 d	PGC-1α↑, Bax↓/Bcl2↑/caspase3↓	Li et al. (2022)
	Sprague-Dawley rats	*Astragalus* injection	4000 mg/kg/d	7 d	MCP-1↓; TGF-β1/Smad2/3↓	Li et al. (2014b)
	HMrSV5 cells	*Astragalus*	0–800 mg/ml	0–72 h	GSK3β/β-catenin complex↑/β-catenin↓/EMT↓; Smad7↑/β-catenin↑/EMT↓	Yu et al. (2018)
	Sprague-Dawley rats		4000 mg/kg/d	35 d		
	Rat peritoneal mesothelial cells (RPMCs)	*Astragalus* injection	2 g/ml	0–48 h	TGF-β1↓/NADPH Oxidase Subunit p67phox↓/ROS↓/EMT↓	Liu et al. (2014)
Renal fibrosis	C57BL/6J mice	*Astragalus* polysaccharides	200 mg/kg every 2 days	21 d	TGF-β1/ILK↓	Zheng et al. (2021)
	mice	*Astragalus*	100, 200, 400 mg/kg/d	7 d	TGF-β1/Smad2/3↓/EMT	Shan et al. (2016)
	NRK-52E		10, 20, 40 μg/ml			
	mice	Astragaloside IV	20, 40 mg/kg/day	7 d	TLR4/NF-кB↓	Zhou et al. (2017)
	HK-2 cells		10, 20 μM	48 h		
	C57BL/6 mice	Astragaloside IV	20, 40 mg/kg/d	4 w	p62 phosphorylation/Keap1↑/Nrf2 nuclear translocation↑/ROS↓	Gao et al. (2020)
	HK-2 cells		50, 100 μM	24 h		
	Sprague-Dawley rats	Astragaloside IV	80 mg/kg	12 w	collagen IV↓, FN↓, advanced glycation end products↓, IL-1β↓, IL-18↓	Zhang et al. (2020b)
	Renal fibroblasts	Astragaloside IV	10,50 and 100 μM	48 h	MAPK and NF-κB signaling pathways↓	Che et al. (2015)
	KKAy mice	Astragaloside	40 mg/kg/d	10 w	TGF-β1/Smad2/3↓	Wang et al. (2015)
	Sprague-Dawley rats	Calycosin	5 mg/kg	8 w	Nrf-2↑; IL33/ST2↓	Elsherbiny et al. (2020)
	KK-Ay mice	Astragaloside IV	40 mg/kg/d	12 w	inhibition of mir-21-induced podocyte dedifferentiation and mesangial cell activation	Wang et al. (2018)
	C57BL/6 mice	Astragaloside IV	20 mg/kg	7 d	inhibition of MAPK pathway	Xu et al. (2014)
	db/db mice	Formononetin	25, 50 mg/kg	8 w	sirt1↑/Nrf2 nuclear translocation↑/FN and intercellular adhesion molecule 1–1↓	Zhuang et al. (2020)
Cardiac fibrosis	C57BL/6 mice	*Astragalus* polysaccharides	200 mg/kg/d	5 w	TLR-4/NF-κB p65 signal pathway↓	Liu et al. (2019c)
	Cardiac fibroblasts	*Astragalus* saponin	0, 10, 20 μg/ml	1 h	ROS↓, TGF-β1/Smad2/3↓, TIMP1↑, Smad7↑	Gu et al. (2014)
	Rats	Astragaloside IV	10 mg/kg/d	35 d	oxidative stress↓, anti-ferroptotic action by enhancing Nrf2 signaling	Luo et al. (2021)
	Sprague-Dawley rats	Calycosin	80 mg/kg/d	28 d	PI3K↑/AKT↑/STAT3↓/MMP-9↓	Wang et al. (2022b)
	Cardiac fibroblasts		5 μM	24 h		
	mice	Calycosin	_	_	suppressing TGFBR1 signaling pathways	Chen et al. (2022)
	C57BL/6 mice	Astragaloside IV	100 mg/kg/d	2 w	FAS/FASL↓	Liu et al. (2019b)
	Sprague-Dawley rats	Astragaloside IV	5, 10 mg/kg	10 d	TRPM7 channel↓	Lu et al. (2017)
	Cardiac fibroblasts		1, 10 μM	48 h		
	Sprague-Dawley rats	Astragaloside IV	10 mg/kg/d	10 d	mir-135a↑/TRPM7↓/TGF-β/Smads↓	Wei et al. (2020)
	C57BL/6 mice	Astragaloside IV	40 mg/kg/d	28 d	NOX2↓, NOX4↓, ROS↓	Lin et al. (2019)
	Cardiac fibroblasts	Astragaloside IV	100 μM	24 h	suppressing ROS-mediated MAPK activation	Dai et al. (2017)
	Sprague-Dawley rats	Astragaloside IV	80 mg/kg/d	14 d	inhibition of ROS-mediated CT-1 overexpression	Jia et al. (2017)
	BALB/c mice	Astragaloside IV	100, 200 mg/kg/d	7 d	inhibition of NLRP3/caspase1/IL-18 pathway	Wan et al. (2018)

Studies on the pharmacokinetics of *Astragalus* are rare, a study showed that the primary metabolites and secondary metabolites of Astragali Radix from different origins were very different in mice after oral administration. Therefore, studies on the pharmacokinetics of *Astragalus* need to limit the origin, season, and planting years (Li et al., 2015). Water-soluble extracts of Astragali Radix include flavonoids, saponins, carbohydrates, amino acids, organic acids, and nucleotide derivatives (Li et al., 2015). The water extracts of Astragali Radix contain Calycosin 0.1934 mg/g, formononetin 0.16 mg/g, and Astragaloside IV 0.29 mg/g, the mean half-life (t1/2) was between 1 and 5 h (Shi et al., 2015). In the human body, flavonoids can be absorbed orally, Calycosin and formononetin are important flavonoids, and the major metabolites are glucuronides, such as calycosin-7-*O*-β-D-glucoside, formononetin-7-*O*-β-D-glucoside (Xu et al., 2006). In rats, the oral bioavailability of formononetin is 21.8% (Luo et al., 2018). In rats, up to 170 compounds (23 original constituents and 147 metabolites) were found *in vivo* after oral administration of Astragali Radix total flavonoids, Calycosin-3′-O-glucuronide was identified as the main metabolite of Calycosin. Calycosin and formononetin were widely distributed within rat tissues, including kidneys, lungs, heart, spleen, liver, thymus, and colon, except the brain (Liu et al., 2020).

However, lower plasma concentrations of saponins were observed in rat and human plasma, which may be associated with low bioavailability and intestinal bacterial metabolism (Xu et al., 2006; Shi et al., 2015). The absolute oral bioavailability of Astragaloside IV is only 7.4% and 2.2% in beagle dogs and rats, respectively (Gu et al., 2004; Zhang et al., 2007). After oral administration of *Astragalus* aqueous extract (4 g/kg raw herb), the peak concentration (Cmax) and elimination half-life (t1/2) of Astragaloside IV is 7.99 ± 5.97 ng/ml and 5.09 ± 2.26 h in beagle dogs (Yu et al., 2022). Intravenous administration has greater bioavailability than oral administration, about 50% of Astragaloside IV can be metabolized *in vivo* by intravenous administration in rats (Du et al., 2005). Moreover, Astragaloside IV is rapidly absorbed, metabolized by the liver, and widely distributed in the body. After intravenous administration of Astragaloside IV at a dose of 4 mg/kg for 10 min, Astragaloside IV can be found in the liver, kidney, lung, heart, and spleen in rats, with the highest content in the liver and kidney (Chang et al., 2012). In addition, *Astragalus* polysaccharides also have low bioavailability due to molecular weight and low solubility (Du Y. et al., 2022).

In terms of safety, *Astragalus* may be safe for most adults. In rats, the acute oral median lethal dose was more than 250.00 g/kg BW, and no harmful effects were found in the 90-day oral toxicity test at a dose of 15.00 g/kg BW (Li H. et al., 2021). No significant adverse effects were found in rat and beagle dog models when *Astragalus* extract was administered intraperitoneally or intravenously for three consecutive months. For rats, the safe dosage range is 5.7–39.9 g/kg and for beagle dogs, it is 2.85–19.95 g/kg, which is equal to 70 or 35 times that of human (0.57 g/kg, average BW 70 kg), respectively (Yu et al., 2007). In the human body, after intravenous administration of Astragalosides injection of 200–500 ml for 7 days, only transient adverse events were found, such as raised total bilirubin and rash (Xu et al., 2013).

However, intravenous administration of Astragaloside IV (0.5 and 1.0 mg/kg) affects fetal survival in rats or rabbits (Jiangbo et al., 2009), another study found that intravenous administration of 0.25 mg/kg to 1.0 mg/kg of astragaloside IV inhibited fertility in rats (Xuying et al., 2010). Therefore, *Astragalus* should be used cautiously in pregnant women.

## Effect of Astragalus on fibrotic diseases

**FIGURE 2:**
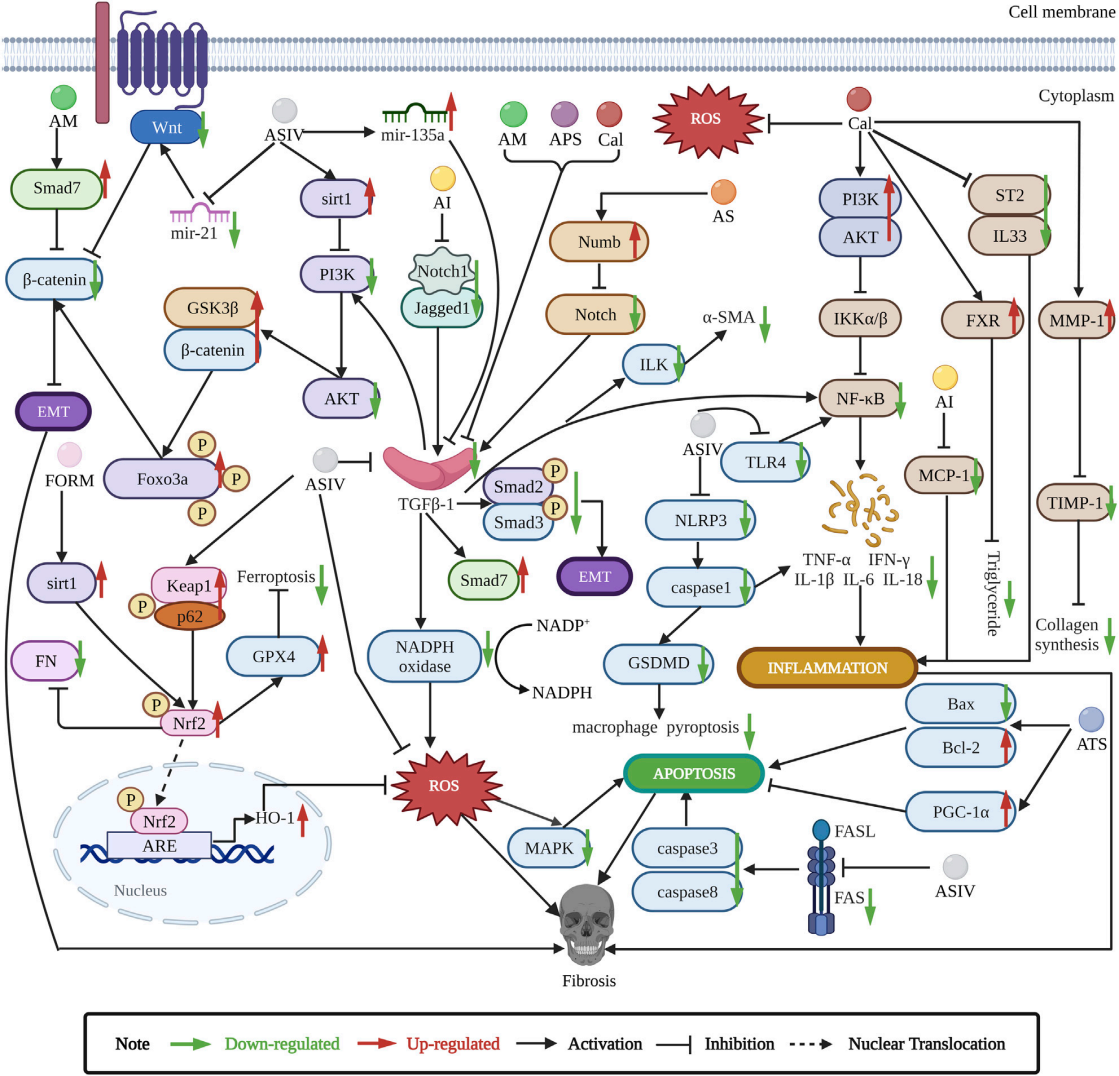
*Astragalus* exerts antifibrosis effects by inhibiting EMT, ROS, TGF-β1/Smads, apoptosis, and inflammation pathways. ASIV is the main antifibrotic component of *Astragalus*. ASIV downregulates mir-21/Wnt/β-catenin/EMT pathway (Wang et al., 2018), TGF-β1/PI3K/AKT/Foxo3a/EMT pathway (Qian et al., 2018), and sirt1 AS/EMT pathway (Qian et al., 2020). ASIV also regulates mir-135a/TGF-β/Smads pathway (Wei et al., 2020). ASIV reduces oxidative stress by upregulating p62/Keap1/Nrf2 pathway (Gao et al., 2020), ASIV enhances Nrf2 signaling to inhibit ROS-induced ferroptosis (Luo et al., 2021). In addition, ASIV can modulate MAPK and NF-κB signaling pathways to inhibit inflammation and apoptosis (Xu et al., 2014; Che et al., 2015; Dai et al., 2017; Zhou et al., 2017). ASIV inhibits NLRP3/caspase1 pathway (Wan et al., 2018; Zhang et al., 2022b). ASIV downregulates FAS/FASL pathway to inhibit apoptosis (Liu et al., 2019b). ASIV also reduces TNF-α and IL-6 expression to inhibit inflammation (Qian et al., 2018). Cal can suppress TGFBR1 signaling pathway (Chen et al., 2022). Cal also modulates PI3K/AKT and IL33/ST2 pathways to suppress inflammation (Elsherbiny et al., 2020; Wang et al., 2022b). Cal activates FXR to reduce triglyceride (Duan et al., 2017). Cal reduces ROS and balances MMP-1/TIMP-1 system to inhibit collagen synthesis (Zhang et al., 2021a). AI inhibits Jagged1/Notch1//TGF-β1/Smads/EMT and TGF-β1/NADPH oxidase/ROS pathways (Liu et al., 2014; Zhou et al., 2016b), AI can reduce MCP-1 expression to inhibit inflammation (Li et al., 2014b). FORM can regulate sirt1/Nrf2/ARE/ROS signaling pathway (Zhuang et al., 2020). AM modulates Smad7/β-catenin/EMT pathway (Yu et al., 2018). AS regulates numb/Notch/TGF-β1 pathway (Yongping et al., 2015). APS inhibits TGF-β1/ILK pathway to reduce inflammation (Zheng et al., 2021). ATS modulates PGC-1α and Bax/Bcl2/caspase3 pathway to inhibit apoptosis (Li et al., 2022).

Here, we summarize the antifibrosis mechanisms of *Astragalus* (Figure 2).

### Anti-hepatic fibrosis effect of Astragalus

In previous studies, the PDGF family was shown to induce hepatic stellate cell activation to aggravate hepatic fibrosis. However, Astragaloside IV can suppress PDGF-BB-induced hepatic stellate cells activation to reduce collagen I, α-smooth muscle actin (α-SMA), and FN deposition by activating the nuclear factor kappa-B (NF-κB) pathway *in vitro* (Chen et al., 2019). In addition, bile stasis causes cholestatic liver fibrosis (Hasegawa et al., 2021), intraperitoneal administration of Astragaloside in a bile duct ligation model of hepatic fibrosis in rats attenuated the development of fibrosis as assessed by collagen deposition, myofibroblast activation, and hepatobiliary function (Yongping et al., 2015). Furthermore, fibrosis leads to changes in cellular structure and mechanical properties (Natarajan et al., 2017), *Astragalus* polysaccharide can increase the stiffness of liver sinusoidal endothelial cells, which may provide a new mechanism for Chinese medicine to fight hepatic fibrosis (Lu et al., 2018). Calycosin can reduce α-SMA in both carbon tetrachloride-induced hepatic fibrosis mice and non-alcoholic steatohepatitis mice, collagen I decrease is also observed in the carbon tetrachloride model, this is related to multiple mechanisms, including inhibiting oxidative stress, matrix metalloproteinase-1/tissue inhibitor of metalloproteinase-1 (MMP-1/TIMP-1) system and activating janus kinase 2- signal transducer and activator of transcription 3 (JAK2-STAT3) pathway, while Calycosin activates farnesoid X receptor (FXR) to attenuate hepatic fibrosis in non-alcoholic steatohepatitis mice (Duan et al., 2017; Zhang et al., 2021a).

### Anti-pulmonary fibrosis effect of Astragalus

In pulmonary fibrosis rats, Astragaloside IV significantly inhibits the TGF-β1/Smad signaling pathway and attenuates extracellular matrix deposition, such as collagen I, collagen III, laminin, hyaluronic acid, hydroxyproline, high-mobility group box1 (Li et al., 2017; Li et al., 2019; Li N. et al., 2021). Besides, EMT plays a vital role in pulmonary fibrosis (Muthuramalingam et al., 2020). The inhibitory effect of *Astragalus* active ingredients on EMT has been confirmed in bleomycin induced-pulmonary fibrosis rats. Astragaloside IV inhibits TGF-β1/phosphoinositide-3-kinase (PI3K)/protein kinase B (AKT)-induced forkhead box O3a (Foxo3a) hyperphosphorylation and down-regulation to reverse EMT (Qian et al., 2018). Furthermore, long noncoding RNAs sirt1 antisense (sirt1 AS) is involved in organ fibrosis, sirt1 AS enhances the stability of sirt1 and increases sirt1 expression, thereby inhibiting EMT in idiopathic pulmonary fibrosis (IPF). Interestingly, Astragaloside IV treatment increases sirt1 AS expression and suppresses EMT (Qian et al., 2020). Additionally, Calycosin suppresses the AKT/glycogen synthase kinase 3β (GSK3β)/β-catenin signaling pathway to inhibit EMT (Liu et al., 2021b), *Astragalus* polysaccharides inhibit TGF-β1/EMT and NF-κB pathway activation (Zhang et al., 2020a). *Astragalus* injection also reduces collagen accumulation and α-SMA protein overexpression in bleomycin-induced-pulmonary fibrosis rats, this protective effects may be related to Jagged1/Notch1 downregulation in the lung (Zhou et al., 2016b).

### Anti-peritoneal fibrosis effect of Astragalus

Monocytes/macrophages are the principal cells in inflammation and monocyte chemoattractant protein (MCP)-1 is an effective chemokine that activates macrophages and promotes monocytes migration into tissue during inflammation (Reinecker et al., 1995; Shi and Pamer, 2011; Oishi and Manabe, 2018). Happily, *Astragalus* injection effectively reduces MCP-1 expression, inhibits monocytes/macrophages recruitment and activation, and suppresses TGF-β1 production in rats submitted to peritoneal dialysis, indicating its mechanisms of anti-peritoneal fibrosis effects may involve both MCP-1 and the TGF-β/Smad pathways (Li et al., 2014b). In addition, EMT is crucial for causing fibrosis and the accompanying decline in peritoneal membrane function (Strippoli et al., 2016; Balzer, 2020). *Astragalus* and *Astragalus* injection can inhibit rat and human peritoneal mesothelial cell EMT *in vitro*. Mechanisms are related to downregulating β-catenin and nicotinamide-adenine dinucleotide phosphate (NADPH) oxidase-dependent formation of ROS (Liu et al., 2014; Yu et al., 2018). Furthermore, *Astragalus* total saponins can promote the peroxisome proliferator-activated receptor gamma co-activator (PGC-1α) in peritoneal fibrosis rats, which increases mitochondrial synthesis to inhibit apoptosis and fibrosis, evidenced by decreased Smad2, α-SMA, caspase3, and Bax (Li et al., 2022).

### Anti-renal fibrosis effect of Astragalus

Nearly all kidney disorders have a common ultimate pathway, and growing renal fibrosis is linked to functional impairment (Nastase et al., 2018). In hypertensive mice, TGF-β1/integrin-linked kinase (ILK) signaling pathway is activated, while it is downregulated after *Astragalus* polysaccharide treatment, as evidenced by reduced IL (interleukin)-1β, IL-6, α-SMA, collagen I, and collagen III (Zheng et al., 2021). Renal tubulointerstitial fibrosis is made worse by EMT. *Astragalus* treatment reduces TGF-β1/Smad2/3 signaling pathway to antagonize tubular EMT and deposition of FN and collagen I, significantly ameliorating renal interstitial fibrosis in a mouse model of unilateral ureteral obstruction (UUO) (Shan et al., 2016). *In vitro*, Astragaloside IV inhibits mitogen-activated protein kinase (MAPK) and NF-κB signaling pathways in mice renal fibroblasts, which reduces α-SMA, FN, and collagen I (Che et al., 2015), which is verified in UUO mice. A UUO mouse model for intraperitoneal injection of Astragaloside IV reduced α-SMA, FN, and collagen IV *in vivo via* suppressing the MAPK pathway (Xu et al., 2014). Recently, Astragaloside IV has been proven to ameliorate renal fibrosis by inhibiting inflammation *via* the toll-like receptor 4 (TLR4)/NF-кB signaling pathway in UUO mice (Zhou et al., 2017). Besides, Astragaloside IV significantly alleviates tubulointerstitial fibrosis by upregulating p62- Kelch-like ECH-associated protein 1 (Keap1)- Nuclear factor erythroid 2-related factor 2 (Nrf2) pathway in tacrolimus treated mice (Gao et al., 2020). A db/db (diabetic) mouse model of diabetic renal fibrosis is improved by formononetin through activating the Nrf2/antioxidant response element (ARE) signaling cascade *via* sirt1 (Zhuang et al., 2020). Astragaloside IV also reduces collagen IV, FN, and CD36 expression in the kidney tissues of diabetic nephropathy rats, thus delaying the process of renal fibrosis (Su et al., 2019; Zhang et al., 2020b). Recent studies also demonstrated that Astragaloside IV functioned an inhibitory role in renal fibrosis by inhibiting mir-192 and mir-21 in animal models, which contributes to suppressing TGF-β1 (Wang et al., 2018; Cao et al., 2019; Mao et al., 2019). In addition, Calycosin regulates both oxidative stress and inflammation process to suppress renal fibrosis in diabetic rats, which is related to Nrf2 upregulation and IL33/ST2 inhibition (Elsherbiny et al., 2020).

### Anti-cardiac fibrosis effect of Astragalus

*Astragalus* polysaccharide could suppress the TLR4/NF-κB p65 signal pathway and protects mice from coxsackievirus B3 (CVB3)-induced virus myocarditis *in vivo* (Liu et al., 2019c). Similarly, Astragaloside IV suppresses the FAS/FASL signaling pathway and protects mice against CVB3-induced myocardial damage and fibrosis *in vivo* (Liu et al., 2019b). Fibrosis is the principal pathological change of radiation-induced heart disease and *Astragalus* saponin decreased collagen I, and TGF-β1 expression of rats’ cardiac fibroblasts *in vitro*, which may be closely related to its antioxidant action (Gu et al., 2014). Calycosin can ameliorate myocardial infarction-induced inflammation and fibrosis *via* activation of PI3K-AKT-IκB kinase α/β (IKKα/β) in heart failure rats and transforming growth factor-beta receptor 1 (TGFBR1) pathways in heart failure mice (Wang et al., 2022b; Chen et al., 2022). Astragaloside IV significantly downregulates the TRPM7 channel to reduce hypoxia-induced cardiac fibrosis, further study verifies that Astragaloside IV inhibits cardiac fibrosis by targeting the mir-135a-TRPM7-TGF-β/Smads pathway in rats and *in vitro*, α-SMA and collagen I are also decreased (Lu et al., 2017; Wei et al., 2020). What’s more, oxidative stress is directly or indirectly involved in cardiac fibrosis (Kong et al., 2014). Fortunately, Astragaloside IV has proven to be effective at regulating ROS through multiple pathways. ROS level is involved in TGF-β-mediated fibrosis and ferroptosis is involved in lipid ROS generation. Study shows that Astragaloside IV has an anti-ferroptotic action by enhancing the Nrf2 signal pathway, which may play a protective role against adriamycin-induced myocardial fibrosis in rats as assessed by collagen I, III, and TGF-β (Luo et al., 2021). Astragaloside IV treatment also relieves NADPH oxidase 2, 4 expression and oxidative stress in cardiomyocytes *in vitro*, leading to decreased myocardial fibrosis (Lin et al., 2019). Astragaloside IV may suppress ROS-mediated MAPK activation and cardiotrophin-1 (CT-1) upregulation to inhibit cardiac fibrosis *in vitro*, as Astragaloside IV reduces isoprenaline-induced rat cardiac fibroblast proliferation and collagen Ι deposition (Dai et al., 2017; Jia et al., 2017). Astragaloside IV also alleviates myocardial fibrosis by suppressing ROS/caspase1/gasdermin D (GSDMD) signaling pathway in mice (Zhang et al., 2022b). Besides, Astragaloside IV exhibits antifibrosis effects through inhibition of the NOD-like receptors family pyrin domain-containing 3 (NLRP3)/caspase1/IL-18 pathway in isoproterenol-induced cardiac fibrosis mice, evidenced by decreased collagen I, collagen III, and TGF-β1 (Wan et al., 2018). Gut microbiota is important to cardiac health, Astragaloside IV may decrease α-SMA expression and ameliorate cardiac fibrosis by increasing the Akkermansia, Defluviitaleaceae_UCG-011, and Rikenella abundance and modulating amino acid metabolism in isoprenaline-induced cardiac fibrosis mice (Du X. Q. et al., 2022).

### Other anti-fibrotic effects of Astragalus

*Astragalus* possesses broad anti-fibrotic effects, *Astragalus* polysaccharide may inhibit TGF-β1 production to manage systemic scleroderma fibrotic disorders in bleomycin-induced scleroderma murine model (Hao et al., 2015). Similarly, Calycosin may reduce α-SMA, collagen I and modulate the TGF-β signaling pathway to inhibit intestinal fibrosis on CCD-18Co cells *in vitro* (Liu et al., 2019a). Astragaloside IV decreases TGF-β2 and FN, collagen I to reduce mouse glaucomatous trabecular meshwork fibrosis *in vivo* and *in vitro* (Kasetti et al., 2021). Astragaloside IV and *Astragalus* polysaccharides promote wound healing and inhibit scar formation *in vivo* (Chen et al., 2013; Zhao et al., 2017). The former process may be related to inducing cell proliferation, cell migration, and angiogenesis (Chen et al., 2012), and the latter process may be related to suppressing excessive inflammation and reducing collagen I, collagen III, and FN deposition (Chen et al., 2012; Luo et al., 2016; Wang et al., 2022a). Although both processes involve collagen deposition, more inflammatory responses can be detected in scar formation, so this two-way regulation may be related to suppressing the inflammatory response.

### Anti-fibrosis effect of herbal formulas containing Astragalus

Compatibility of herbs is one of the advantages of traditional Chinese medicine and is believed to elicit therapeutic effects (Wang et al., 2021). As shown in Table 2, *Astragalus* is often combined with other herbs, such as *Angelica sinensis* (Oliv.) Diels and *Salvia miltiorrhiza* Bunge or in various complex prescription formulas.

**TABLE 2:** Effect of herbal formulas containing *Astragalus* on fibrotic diseases.

Decotion	Contents	Animal/Cell line	Dose	Duration	Disease	Mechanisms	References
Huang Qi Decoction	*Astragalus* 30 g *Glycyrrhiza glabra* L. 5 g	Sprague-Dawley rats	172.76 mg/kg/d	3 w	hepatic fibrosis	α-SMA↓, collagen IV↓, collagen I↓, TNF-α↓, TGF-β1↓, Smad7↑, Notch signaling↓	Zhang et al. (2017)
		WB-F344 cell line	800 μg/ml	7 d	hepatic fibrosis	targeting Numb gene	Xu et al. (2020)
Yangfei Huoxue Decoction	*Astragalus* 20	Sprague-Dawley rats	4.59, 9.18, 18.36 g/kg/d	28 d	pulmonary fibrosis	Notch↓	Chen et al. (2020)
	*Glehnia littoralis* (A.Gray) F.Schmidt ex Miq. 20						
	*Schisandra chinensis* (Turcz.) Baill. 8						
	*Salvia miltiorrhiza* Bunge 15, *Reynoutria japonica* Houtt. 15, *Conioselinum anthriscoides ‘Chuanxiong’* 12						
	*Euonymus alatus* (Thunb.) Siebold 12						
	*Astragalus* 20 g	Sprague-Dawley rats	4.59, 9.18, 18.36 g/kg/d	28 d	pulmonary fibrosis	VEGF and IL-1β↓	Liu et al. (2019d)
	*Glehnia littoralis* subsp. Littoralis 20 g						
	*Schisandra sphenanthera* Rehder and E.H.Wilson 8 g						
	*Salvia miltiorrhiza* Bunge 15g, *Reynoutria japonica* Houtt. 15g, *Conioselinum anthriscoides ‘Chuanxiong’* 12 g						
	*Euonymus alatus* (Thunb.) Siebold 10 g						
Danggui Buxue Decoction	*Astragalus*, *Angelica sinensis* (Oliv.) Diels	Sprague-Dawley rats	4, 8, 16 mg/kg/d	4 w	pulmonary fibrosis	NADPH oxidase-4↓/oxidative stress↓	Zhao et al. (2015)
	*Astragalus*, *Angelica sinensis* (Oliv.) Diels	Wistar rats	16, 32, 64 mg/kg/d	4 w	pulmonary fibrosis	MMP-9↓, TIMP-1↓	Gao et al. (2012)
	*Astragalus* 30 g	Sprague-Dawley albino rats	10, 1000 mg/kg/d	8 w	renal fibrosis	FN↓, collagen IV↓, TIMP-1↓, TGF-β1↓, MMP-9↑	Wei et al. (2012)
	*Angelica sinensis* (Oliv.) Diels 15 g						
	*Conioselinum anthriscoides ‘Chuanxiong’* 15 g						
	*Astragalus*, *Angelica sinensis* (Oliv.) Diels	C57BL/6J mice	0.5 ml	21 d	renal fibrosis	inhibition of MAPK, PI3K-AKT and TNF signaling pathways	Yuan et al. (2022)
	*Astragalus*, *Angelica sinensis* (Oliv.) Diels	Sprague-Dawley rats	9 g/kg/d	14 d	renal fibrosis	Suppressing NLRP3 Inflammasome	Wang et al. (2016)
Drug combinations	*Astragalus*, *Salvia miltiorrhiza* Bunge	Sprague-Dawley rats	60, 120, 240 mg/kg	4 w	hepatic fibrosis	inhibition of TGF-β/Smad/Wnt pathway	Cao et al. (2020)
	*Astragalus*, *Salvia miltiorrhiza* Bunge	C57BL/6 mice	8.4 g/kg/d	6 w	renal fibrosis	regulation of gut-kidney axis	Han et al. (2021)
	*Astragalus*, *Salvia miltiorrhiza* Bunge	Sprague-Dawley rats	20 mg/kg	8 w	myocardial fibrosis	regulation of protein kinase D1 protein	Mao et al. (2015)
	*Astragalus*, *Salvia miltiorrhiza* Bunge	Sprague-Dawley rats	5 g/kg/day	4 w	myocardial fibrosis	improving tissue energy metabolism, promoting myocardial cell proliferation, maintaining circulatory system homeostasis, inhibiting inflammatory response and oxidative stress	Zhang et al. (2021b)
	*Astragalus* and *Salvia miltiorrhiza* Bunge extract ointment	Rabbits	0.94, 1.88, 3.76%; w/w	7 w	hypertrophic scar	TGF-β/Smad4↓, Smad7↑	Wu et al. (2014)

Huang Qi Decoction prevents cholestatic liver fibrosis by inhibiting the Numb/Notch signal pathway in rats (Zhang et al., 2017; Xu et al., 2020). Yangfei Huoxue Decoction downregulates vascular endothelial growth factor (VEGF), IL-1β, and Notch signal pathways to prevent bleomycin-induced pulmonary fibrosis in rats (Liu et al., 2019d; Chen et al., 2020). Danggui Buxue Decoction was found to have anti-multiorgan fibrosis effects in rats model. For example, Danggui Buxue Decoction inhibits MMP-1, 9, TIMP-1 and downregulates the level of oxidative stress in lung tissue (Gao et al., 2012; Zhao et al., 2015). Danggui Buxue Decoction prevents renal fibrosis through decreasing TIMP-1, TGF-β1 gene, NLRP3 inflammasome expressions and increasing MMP-9 gene expression, leading to decreased collagen IV, collagen I, FN deposition and α-SMA expression in rats (Wei et al., 2012; Wang et al., 2016). What’s more, Danggui Buxue Decoction could prevent renal fibrosis by suppressing the tumour necrosis factor (TNF), MAPK, and PI3K-AKT signaling pathways in UUO mice (Yuan et al., 2022).

The combination of *Astragalus* and *Salvia miltiorrhiza* Bunge is also commonly used in fibrotic diseases. Studies have proven this combination has favorable therapeutic efficacy in hypertrophic scar (Wu et al., 2014), liver fibrosis (Cao et al., 2020), cardiac fibrosis (Mao et al., 2015; Zhang et al., 2021b), renal fibrosis (Han et al., 2021).

## Conclusion and future perspectives

Fibrosis causes considerable morbidity and mortality with no safe and effective treatment, and its mechanisms are poorly understood. *Astragalus*, with significant antifibrosis activities, has been used in traditional Chinese medicine for thousands of years, and its predominant components are saponins, flavonoids, and polysaccharides. This review summarizes the different anti-fibrotic effects of *Astragalus* and its anti-fibrotic components, including liver, pulmonary, peritoneal, renal, and cardiac fibrosis. Its active anti-fibrotic components are Calycosin, Astragaloside IV, *Astragalus* polysaccharides, and formononetin. Major mechanisms are inhibition of EMT, ROS, TGF-β1/Smads, apoptosis, and inflammation pathways. As shown in Table 1 and Figure 2, the inhibition of TGF-β1 may be the primary anti-fibrotic mechanisms of *Astragalus*. Astragaloside IV plays the main anti-fibrotic effects among the four active components. Astragaloside IV, Calycosin, and *Astragalus* polysaccharides can inhibit TGF-β1 to exert anti-fibrotic effects. In addition, Calycosin could inhibit ROS, EMT, and inflammation to exert anti-fibrotic effects, *Astragalus* polysaccharides could inhibit EMT and inflammation to exert anti-fibrotic effects, while formononetin plays an anti-fibrotic role by reducing ROS. We also review formulas containing *Astragalus* with anti-fibrotic effects, in which *Astragalus* and *Salvia miltiorrhiza* Bunge, *Astragalus* and *Angelica sinensis* (Oliv.) Diels are the most commonly used combinations.

Traditional Chinese medicine has the advantages of “multiple ingredients, multiple targets and less side effects” (Wang et al., 2019). Reasonable compatibility of medicinal herbs can increase effectiveness. However, there are still some questions, *Astragalus* contains more than 200 components, the pharmacology of them have not been fully elucidated, simultaneous analysis of two kinds of components is rare (Li et al., 2014a), and even the anti-fibrotic effects of the four active components are still incompletely understood despite extensive studies. Meanwhile, there are over 2000 *Astragalus* species (Li et al., 2014a), and amounts of main components vary in different species, even in the same species among locations and years (Ma et al., 2002; Ma et al., 2022). *Astragalus* is often used in combination with multiple drugs in treating fibrotic diseases. Considering the interference of other components, combining active components into new formulations may be a promising way to develop new drugs (Wu et al., 2014; Mao et al., 2015). For example, Astragaloside I, Calycosin, and levistilide A may be the three main bioactive components in Danggui Buxue Decoction, their combination exerts anti-liver fibrosis effects in mice (Guo et al., 2018). The combination of Astragaloside IV and ferulic acid can improve pulmonary and hepatic fibrosis in animal models (Zhao et al., 2020; Tong et al., 2021). Astragaloside IV combined with Ginsenoside Rg1 ameliorates renal fibrosis in rats with diabetic nephropathy (Du et al., 2018). However, fundamental and therapeutic studies are needed for optimal dose ratio and interactions between active components. More importantly, the oral bioavailability of *Astragalus* is limited, and its pharmacokinetics have not been fully clarified. It is feasible to use its active components as alternations, but their bioavailability is still worthy of attention. Taking Astragaloside IV as an example, its derivative LS-102 has higher bioavailability than Astragaloside IV, with definite efficacy and high safety (Qing et al., 2019). Derivatizations, modifications, co-administration, and nanotechnology can significantly improve the oral bioavailability of drugs. It is suggested that future studies should use these methods to improve the active components’ bioavailability (Zhu et al., 2022). In additon, Astragaloside IV is proven to ameliorate cardiac fibrosis in mice *via* modulating gut microbiota and fecal metabolites (Du X. Q. et al., 2022), given that oral administration is the main way for *Astragalus*, gut microbiota may be involved in fibrotic diseases, how *Astragalus* interacts with gut microbiota in fibrotic diseases is worthy of further study (Bajaj, 2019; Rayego-Mateos and Valdivielso, 2020; Gong et al., 2021). In recent years, multi-omics technology has been developing rapidly, providing new means for traditional Chinese medicine. It is expected to have a deep understanding of the pharmacokinetics and anti-fibrosis mechanisms of *Astragalus* by using multi-omics technology (Guo et al., 2020). Although *Astragalus* is generally considered non-toxic, the interaction between its active components and western medicine still deserves attention, and more research is needed.

Previous small clinical trial show that *Salvia miltiorrhiza* Bunge and *Astragalus* could improve liver fibrosis (Tan et al., 2001). In recent years, there are many clinical trials about *Astragalus* and its active components concentrated on immunomodulatory function (Brush et al., 2006; Latour et al., 2021), anticancer effects (Guo et al., 2012; Hsieh et al., 2020) and cardioprotective effects (Li et al., 2018). Therefore, large sample, multi-center, long-period studies to confirm the anti-fibrotic effects of *Astragalus* are highly warranted.

In conclusion, *Astragalus* is a promising anti-fibrotic drug, and its main anti-fibrotic components are Calycosin, Astragaloside IV, *Astragalus* polysaccharides, and formononetin. Future research should concentrate on these active components to confirm anti-fibrotic effects.

## Abbreviations

AM, *Astragalus mongholicus* Bunge; ASIV, astragaloside IV; APS, *Astragalus* polysaccharides; AI, *Astragalus* injection; Cal, calycosin; ATS, *Astragalus* total saponins; AS, *Astragalus* saponin; FORM, formononetin (Figure 2 was created with BioRender.com).
